# Supplementary material for: Spatial Collinearity Constrains Multivariate Molecular‐Enriched Network Estimation
Source: Hum Brain Mapp. 2026 Sep 20;47(14):e70645. doi: 10.1002/hbm.70645 (PMC13590927; doi:10.1002/hbm.70645)
Supplement: Supplementary file 1 [file HBM-47-e70645-s001.pdf]

Spatial collinearity constrains multivariate molecular-enriched network estimation

Supplementary materials

Timothy Lawn[†], Johan Nakuci, Steve CR Williams, Federico Turkheimer, Mitul A. Mehta

† tim.lawn@psy.ox.ac.uk

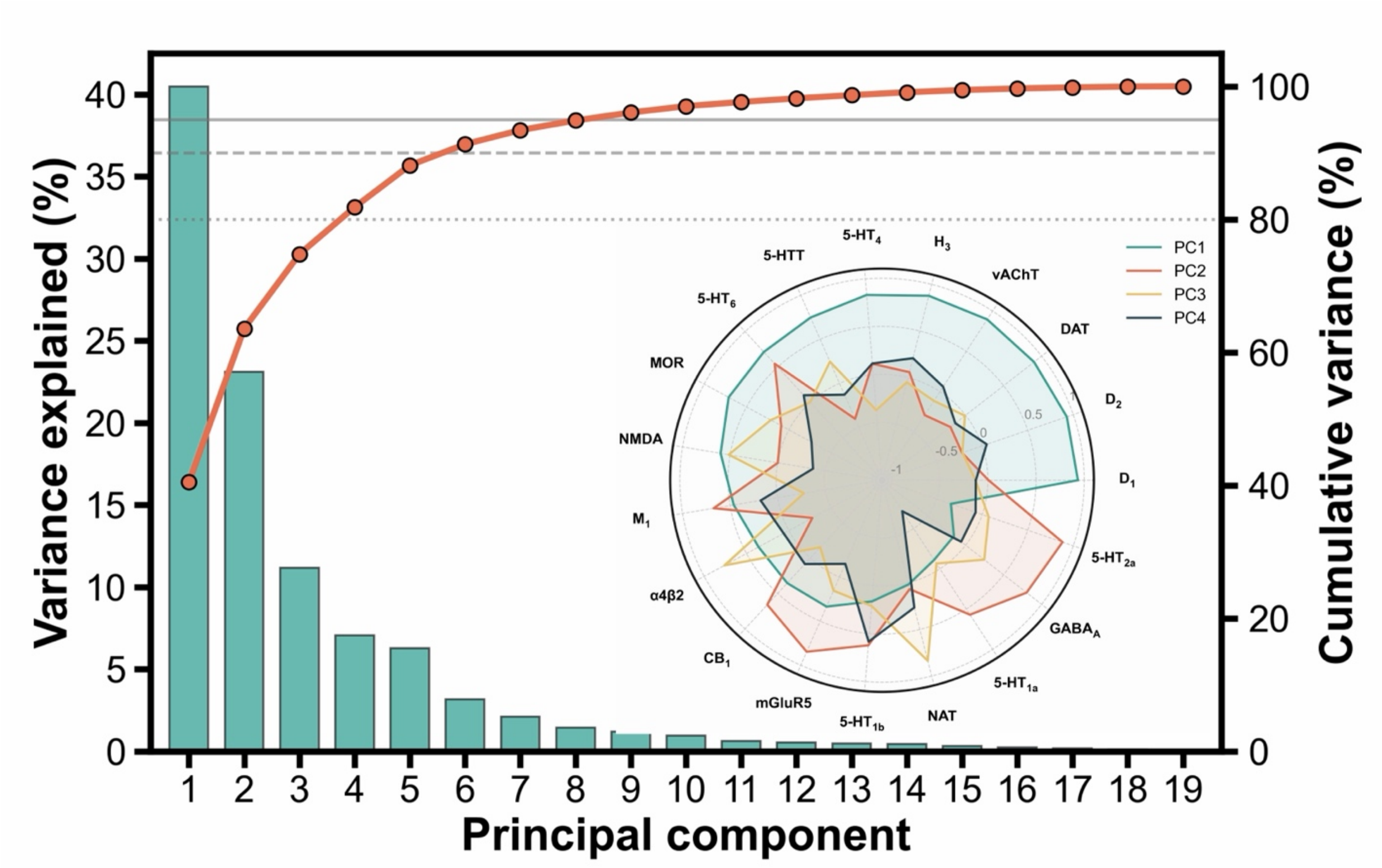

Supplementary Figure 1 | Principal component analysis revealed the effective dimensionality of PET maps. Bars show individual variance explained per component. Orange line shows cumulative variance. Horizontal lines indicate 80% (dotted), 90% (dashed), and 95% (solid) thresholds. Approximately four components account for 80% of the total variance across receptor and transporter maps. This highlights the spatial redundancy in the PET maps. The first four component loadings are shown for each receptor in the radar plot inlay. PC1, the dominant axis of variation, shows a subcortical-cortical contrast: maps with basal-ganglia-concentrated distributions (D_1, D_2, DAT, vAChT) load positively, while predominantly cortical maps (5-HT_{2A}, $GABA_A$) load negatively.

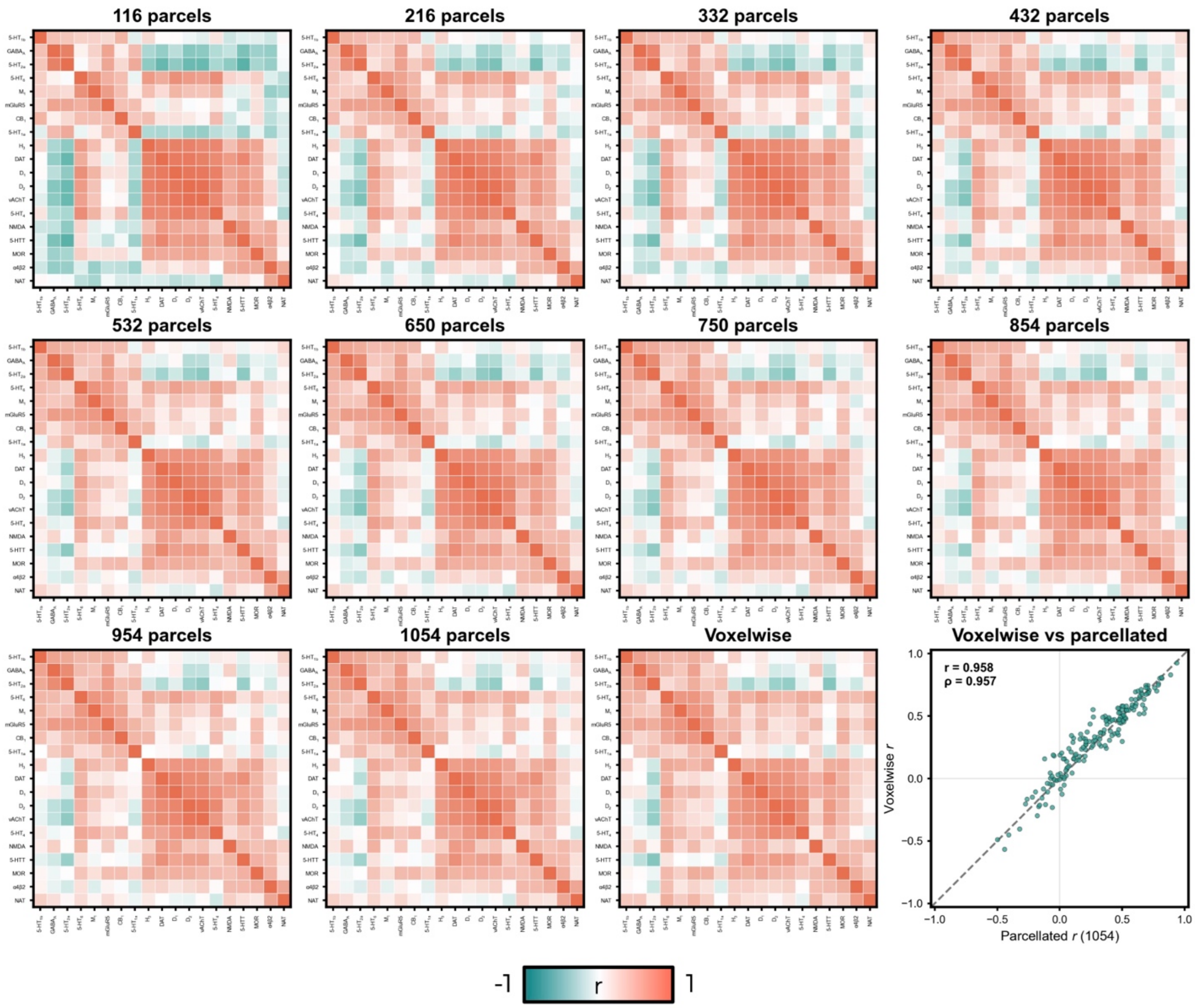

Supplementary Figure 2 | The inter-receptor correlation structure is stable from coarse parcellations through to voxelwise resolution. Pearson correlation matrices among the 19 PET receptor and transporter maps computed at each of the ten parcellation scales (116-1,054 parcels) and at 2mm isotropic voxelwise resolution. Receptor ordering is identical across panels based on the hierarchical clustering solution shown in Figure 2A. The final bottom-right panel plots the vectorised upper triangle of the voxelwise matrix against that of the finest parcellation (1,054 parcels) where each point is one of the 171 unique receptor pairs and the dashed line is the identity line ($r = 0.958$, $\rho = 0.957$). The block structure is already evident at the coarsest scale and changes little beyond 532 parcels, with the voxelwise solution closely matching the parcellated one

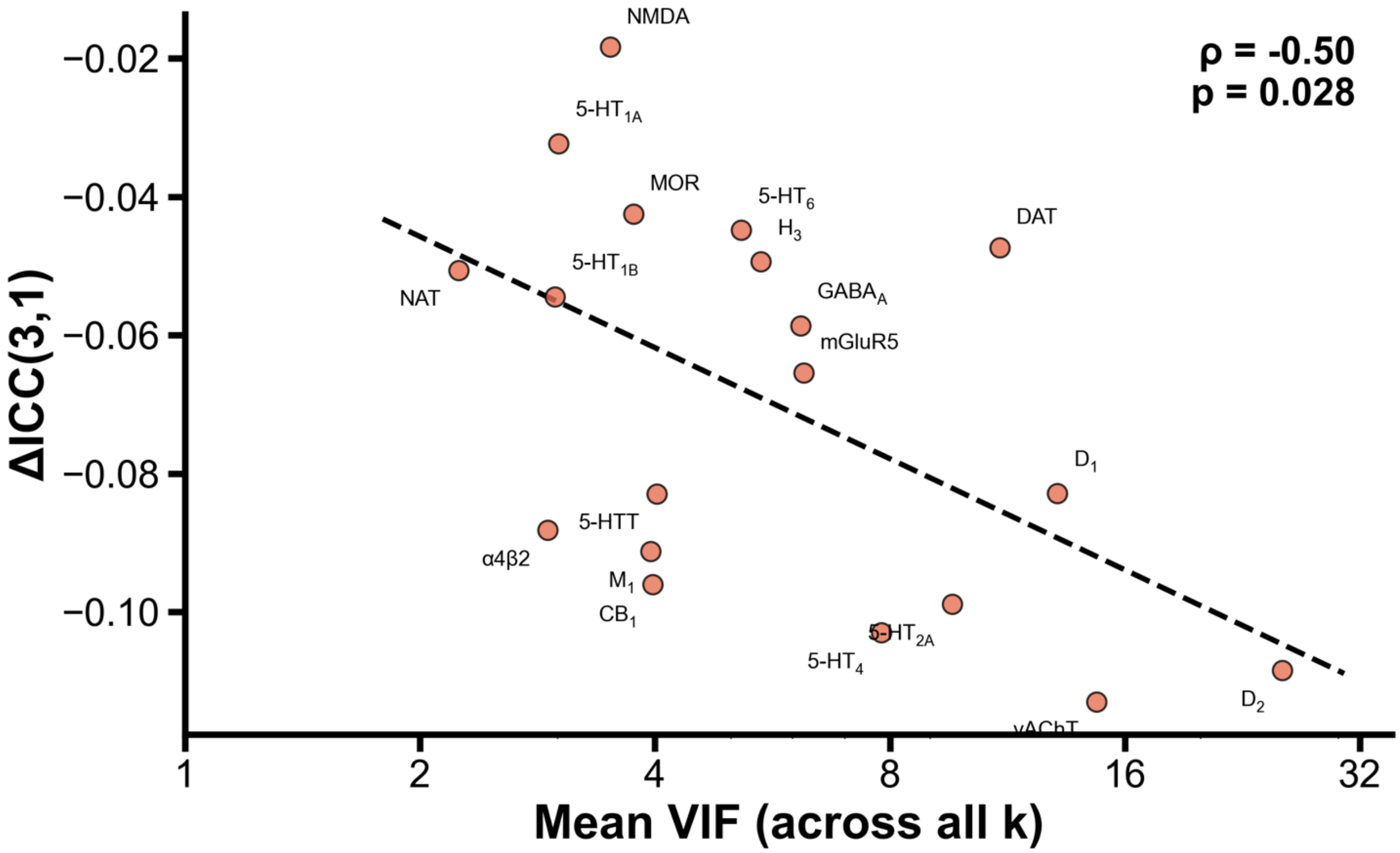

Supplementary Figure 3 | Receptor-level correspondence between collinearity susceptibility and reliability degradation. Each point represents one of the 19 receptors, plotting its grand mean VIF against its grand mean ΔICC, both averaged across model sizes (k = 2 to 19) with each k weighted equally. These per-receptor VIF values use the same k-weighted averaging as the ΔICC, so they differ slightly from the combination-weighted ordering in Figure 2D. The dashed line shows an OLS fit on $\log_{10}$(VIF). Receptors with the most spatially overlapping distributions (e.g., D_2, vAChT, D_1) cluster at the high-VIF, low-ΔICC corner, while spatially distinctive receptors (e.g., NAT, 5-HT_{1A}) are relatively protected. Spearman ρ = -0.50, p = 0.028, n = 19 receptors. Schaefer 400 + Tian S2 parcellation (432 parcels).

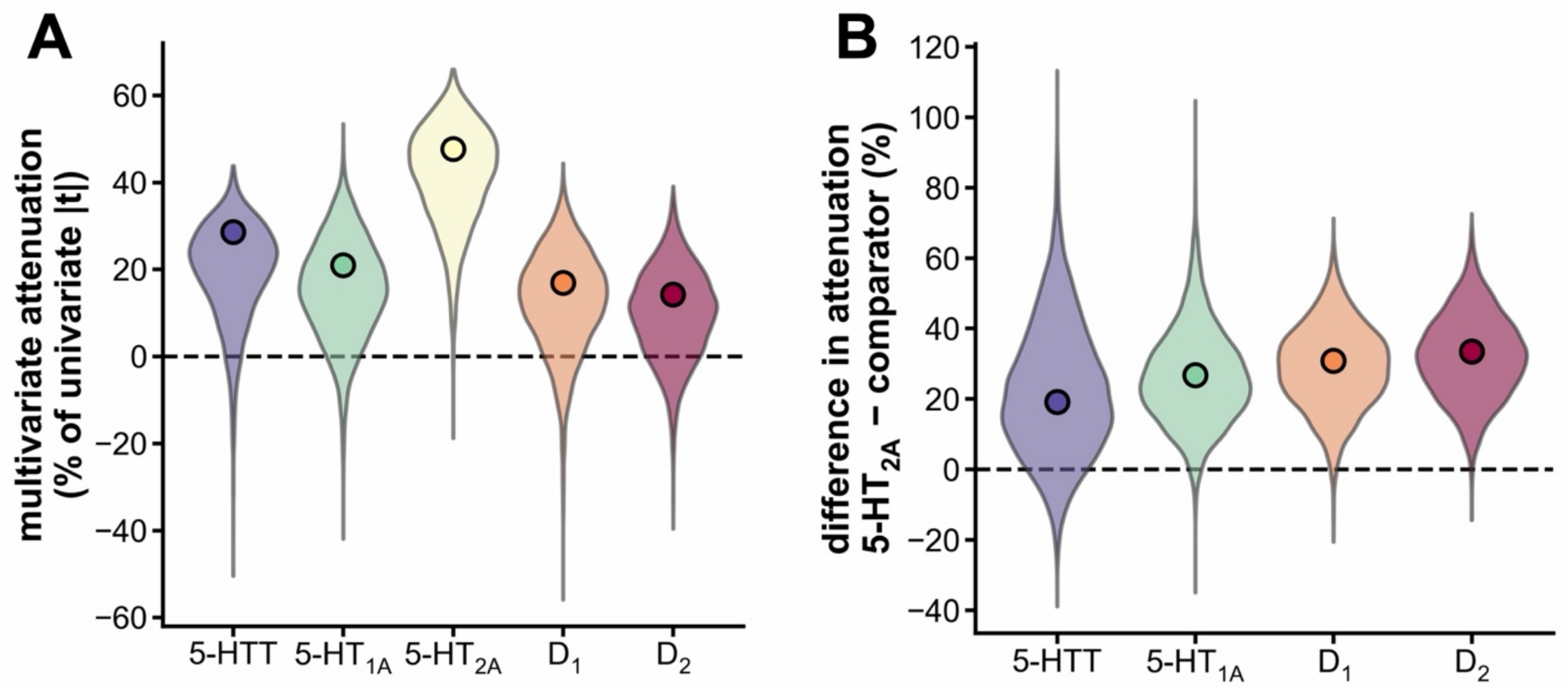

Supplementary Figure 4 | Uncertainty in the multivariate attenuation of LSD drug effects. Participant-level bootstrap (5,000 resamples of the 15 participants, with the same resample applied to both pipelines and all receptors within an iteration). Paired t-maps (LSD minus placebo) were recomputed within each resample and attenuation calculated as the proportional reduction in mean |t| from the univariate to the multivariate pipeline. **(A)** Violins show the bootstrap distributions with the observed values overlaid as points. 95% percentile intervals excluded zero for 5-HT_{2A} (47.7%, 15.4 to 59.2) but not 5-HTT (28.6%, −7.5 to 36.0), 5-HT_{1A} (21.0%, −8.0 to 36.0), D_1 (16.9%, −11.8 to 32.2) or D_2 (14.2%, −11.7 to 28.3). **(B)** Difference in attenuation between 5-HT_{2A} and each comparator, paired within iteration. 5-HT_{2A} attenuated more than every comparator in 90 to 99% of resamples, and intervals excluded zero for D_2 (+8.8 to +55.0), D_1 (+5.4 to +51.8) and 5-HT_{1A} (+2.4 to +56.5) but not 5-HTT (−9.7 to +61.9). Dashed lines indicate no attenuation (A) and equal attenuation (B).

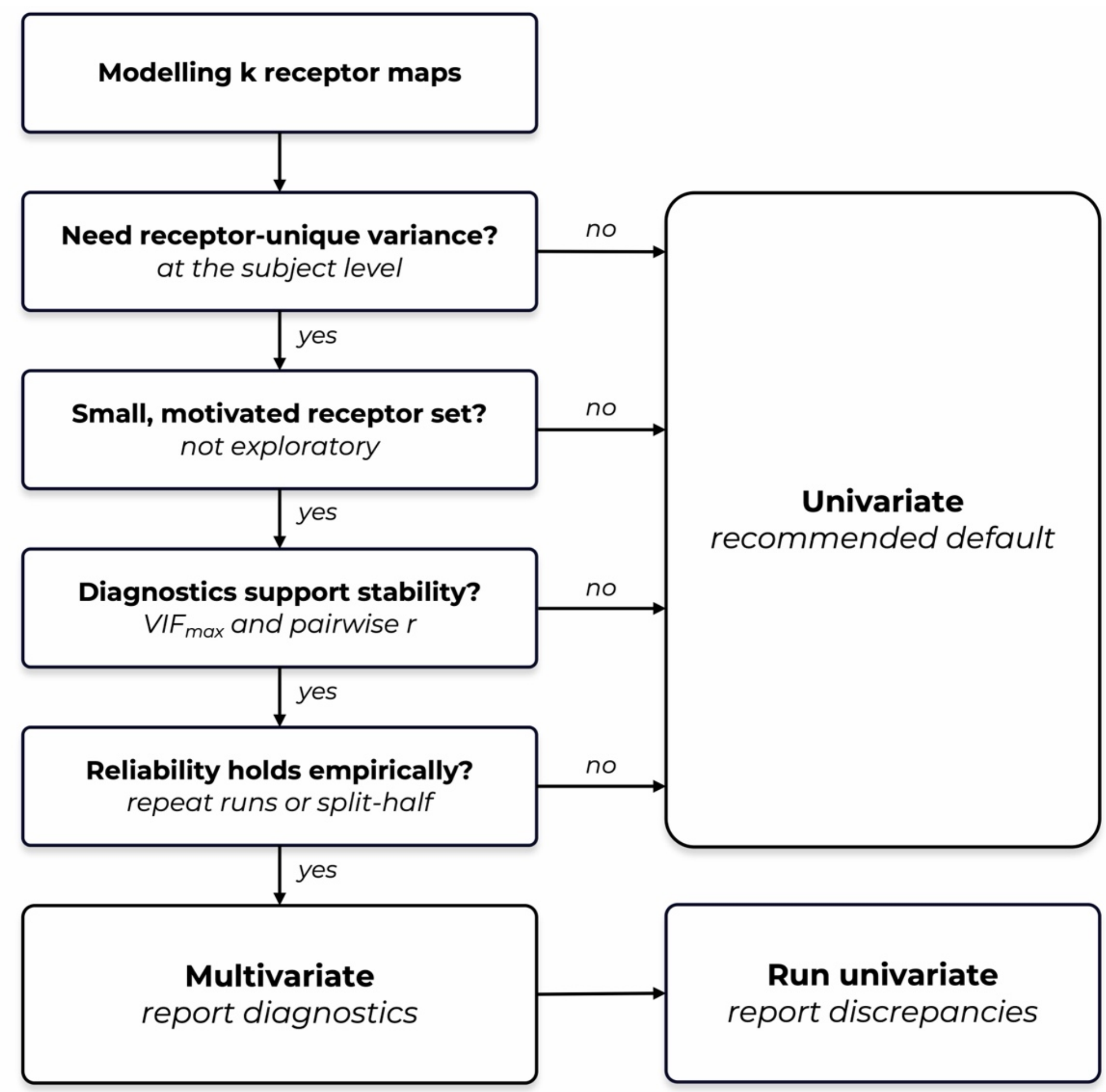

Supplementary Figure 5 | A decision framework for pipeline selection in molecular-enriched network analyses. Univariate modelling is the recommended default. The downward path traces the conditions under which a multivariate model may be justified, along with the checks that should accompany it, and terminates in the univariate pipeline being run alongside so that the two can be compared. Reporting both provides complementary information on upper and lower bounds on the receptor-specific contribution, as done by van den Bosch and Cools, (2026). We suggest the maximum VIF value across receptors in a model (VIF_{max}), or the VIF value of each receptor, be reported instead of mean VIF, which may obscure collinearity affecting individual receptors. We deliberately do not specify numeric thresholds for these diagnostics given that reliability degraded continuously with collinearity rather than beyond an identifiable breakpoint, and receptors with modest VIF were not protected from substantial reliability loss. Conventional thresholds such as VIF>5 are useful to flag the need for caution rather than to license multivariate modelling below them. We emphasise that these recommendations are based on the results of the present manuscript, which comprise one test-retest dataset and one pharmacoimaging use-case. They should be read as a starting point rather than a settled protocol. How these pipelines behave across other drugs, populations, receptor sets, and analytic goals remains an open empirical question.

Supplementary Discussion: Dimensionality reduction and the interpretation of molecular-enriched networks

A natural suggestion in response to the collinearity within REACT models is to reduce the receptor design matrix to a smaller set of orthogonal dimensions, most commonly by principal component analysis (PCA). The resulting components are uncorrelated by construction and the variance inflation and estimation instability we characterised in the multivariate REACT models are therefore eliminated. We agree with the underlying statistical premise of this suggestion and with the broader premise that some questions concern sets of systems acting in concert rather than a single molecular target. We nonetheless consider PCA poorly suited as a general solution for REACT and unpack the reasons here at greater length than the main manuscript discussion permits. We structure this across our three main objections:

(i) The selection of maps changes the results.

Components are determined by the maps chosen to derive them. A component recovered in one study need not correspond to any component recovered in another. This is related to the issue we raised regarding multivariate REACT models. Because modelling receptors together changes their networks (as exemplified in figure 4B), multivariate models introduce a level of arbitrariness to results which become contingent on the original receptor set chosen. Individual receptor maps in univariate models, whatever their limitations, denote the same biological target across every study that uses them. Components and multivariate networks do not, and results expressed in terms of them are correspondingly difficult to compare or analyse across studies.

(ii) The number of components changes the results.

The principal components of a receptor design matrix are orthogonal linear combinations of the original maps, and because that transformation is invertible, the full set of components spans exactly the same space as the full set of maps. A REACT model containing all components is therefore a reparameterisation of the multivariate model rather than an alternative to it, with identical fitted values and coefficients differing only by a fixed rotation. The low variance inflation factors of such a model are a property of the basis chosen rather than evidence that the shared spatial variance among receptors has been resolved, and any attempt to express the result in receptor terms recovers it. PCA only becomes a distinct model once the components are truncated. However, the ordering PCA imposes works against the purpose of distinguishing receptor contributions. Components are ranked by the variance they capture, so the leading components describe the dominant axes of covariation among the maps while the trailing components describe the finer distinctions between them. Because two spatially similar receptors differ only slightly, the direction along which they differ is by construction a low-variance one and is more likely to be discarded. Truncation therefore preferentially removes the information required to attribute an effect to one receptor rather than another, buying stability at the cost of receptor specificity.

(iii) Components are unlikely to be consistently biologically meaningful.

The fact that we can derive individual PET map loadings for each component does not restore the interpretability of the univariate REACT models. REACT's first stage regresses the BOLD signal at each timepoint onto the receptor maps across space, yielding a timeseries whose value at each TR reflects the extent to which the instantaneous spatial configuration of

BOLD activity conforms to that receptor's distribution. The interpretability of this depends entirely on the regressor being a spatial pattern the BOLD signal can plausibly express at a given timepoint, which requires it to be a meaningful biological distribution. The use of the distribution of receptors (especially in the case when we expect changes in accordance with those receptors such as acute drug challenge) provides this biological meaning in REACT. However, PCA uses the covariance among multiple PET maps to produce a weighted composite which may or may not correspond to any meaningful spatial pattern the BOLD signal actually expresses. In other words, one of the most fundamental underlying premises of REACT is potentially violated by constructing complex composite spatial patterns derived from multiple receptors. In short, running REACT on PCA components is not analytically or inferentially equivalent to estimating each receptor's network directly.

None of this is to say that modelling receptors in groups is never appropriate. Where a research question genuinely concerns a set of systems acting in concert rather than a specific molecular target, a grouped model could be more meaningful than either extreme we consider here. Such grouping may be better constrained by hypothesis than by purely data-driven combination of the maps themselves. How biologically meaningful composite spatial patterns might be derived from multiple PET maps certainly warrants future investigation.

References

van den Bosch, R., Cools, R., 2026. Validating molecular target-enriched fMRI for disentangling drug effects on dopamine. Imaging Neuroscience 4, IMAG.a.1139. https://doi.org/10.1162/IMAG.a.1139
